# Interaction Mechanisms in «Portland Cement—Functional Polymer Mineral Additives» Binder Produced by Different Methods

**DOI:** 10.3390/ma18133178

**Published:** 2025-07-04

**Authors:** Svetlana Bondarenko, Mikhail Lebedev, Valeria Strokova, Irina Markova, Natalia Kozhukhova, Nikita Lukyanenko, Danil Potapov

**Affiliations:** 1 Department of Material Science and Material Technology, Belgorod State Technological University Named After V.G. Shukhov, 308012 Belgorod, Russia; sveta-zolotykh@yandex.ru (S.B.); vvstrokova@gmail.com (V.S.); irishka-31.90@mail.ru (I.M.); niklu03@gmail.com (N.L.); potapovdanil420@gmail.com (D.P.); 2 Department of Road Construction Materials and Chemical Technologies, Moscow Automobile and Road Construction State Technical University, 125319 Moscow, Russia; michaell1987@yandex.ru

**Keywords:** Portland cement, complex mineral binder, polymer–mineral additive, combined grinding method, structure formation

## Abstract

The construction industry is the main consumer of mineral resources. At the same time, the Portland cement (PC) industry occupies a leading position, using expensive, high-quality raw materials. This is due to the high rate of construction in different areas (industrial, civil, road construction, etc.). The widespread application of PC is due primarily to the strength and durability of composite materials based on it. Taking into account their specific purpose, PC-based composites are usually optimized to achieve specified characteristics and rational use of raw materials. To reduce PC consumption and justify the possibility of its use in complex binders, this manuscript analyzes the composition of a functional polymer–mineral additive; the nature and mechanisms of its interaction with PC depend on the method of introducing the additive (dry mixing/joint grinding of the clinker–gypsum mixture with the additive at the stage of binder preparation). Based on the data of XRD, IR, and SEM analysis, as well as taking into account patent information, the composition of the additive was clarified. The combined application of the above methods allowed us to establish the uniformity of the additive distribution in the binder depending on the introduction method and to evaluate the effect of each additive component and its mutual impact on the processes occurring during cement hydration. As a result, it was established that the most effective introduction method is combined grinding. A phenomenological model of the structure formation of additives containing cement paste is proposed. The binder production by the combined grinding method promotes the intensification of the processes occurring during hydration, as evidenced by the data of qualitative and quantitative XRD, IR, and DTA analysis, differential scanning calorimetry (DSC), and TGA analysis.

## 1. Introduction

The most popular material used in construction is Portland cement (PC), used to produce different types of concrete, mortar, wall materials, and road construction materials. High demand for building composites and the requirements imposed on them make it relevant to search for methods to reduce cement costs and improve their physical and mechanical properties. One of the most effective ways is to use additives, which reduce the consumption of expensive mineral resources for cement production and give special properties to final cement-based composites.

Lower consumption of cementitious components together with ensuring the required physical and mechanical characteristics of the final composites is of great interest for road construction. The use of composite binders, which imply the use of various mineral components for partial replacement of PC and polymer additives, is part of the Strategy for the Development of Innovative Activities in the Field of Road Facilities for the Period 2021–2025 and allows for the successful utilization of technogenic raw materials.

At the same time, according to Russian Standard GOST R 70196-2022 [1], large-tonnage waste such as reactive fly ash or metallurgical slags can be used for PC replacement. The use of such binders can significantly improve the properties of reinforced clay soils, as well as ensure the use of clay overburden for construction, and, thereby, solve the problem of accumulation of substandard raw materials on the territory of mining enterprises. In this regard, Russian scientists [2,3,4] and scientists from other countries [5,6] are actively engaged in research devoted to additional cements.

Additives applied in composite binders differ in component composition, consistency, dispersion and, most importantly, functional purpose. There are additives that regulate the properties of ready-to-use concrete and mortar mixtures (plasticization, water-reduction, stabilization, porization), regulate the kinetics of cement paste hardening, increase strength and frost resistance, and provide concrete and mortar with special properties (anti-frost, water-repellent), etc. [7]. A special place is occupied by such mineral additives used in cement in an amount of 10 to 50% or more, as inert and active fillers with hydraulic activity or pozzolanic properties [8]. Regardless of the special functions performed in cement, the additives have a chemical and (or) physical effect on the hydration processes of clinker minerals of PC, the formation of new phases, and the structure formation of the cement paste. Today, complex polyfunctional modifiers are increasingly used, influencing several characteristics at once, usually unrelated to each other. In this case, it is possible to significantly enhance the effect achieved by introducing a one-component additive [9,10,11,12,13,14,15,16,17,18,19,20].

For example, in [11] it was established that the addition of complex chemical additives of the new generation of the Relaxol KDZh-3 series to cement contribute to the creation of new formations that crystallize in a finely dispersed form and clog pores and capillaries in the PC paste, compacting and strengthening its structure. The introduction of the KDZh-3 additive into expanded clay concrete allows increasing its density by 8–10% and strength by 40%. In [12], the effectiveness of the complex polyfunctional additive D5 was experimentally proven, which contributes to the improvement of the technological, physical-mechanical and operational characteristics of concrete, since this additive has a water-reducing, cement-saving, and plasticizing effect.

The key advantages of complex additives are confirmed not only by the results of laboratory studies, but also by production tests [21,22,23]. However, the main disadvantage of ready-to-use complex additives, which complicates their integration into the technological process, is the significant variation in the composition and properties of the raw mixture, especially when used in road construction, and the lack of information on composition. As a rule, information on the properties of additives is of an advertising nature and is not always sufficiently objective. This is explained by the fact that the results of scientific research on the effect of certain components of the additive on the properties of cement have commercial value and are not disclosed in scientific and technical literature. In addition, this slows down progress in the search for new effective and cheap complex additives applicable to PC and organomineral clay composites based on it. One example of a complex polyfunctional additive is a polymer–mineral additive (PMA). According to [24], the efficiency of PMA in PC added by three different methods was investigated, and the physical and mechanical properties (Table 1) and morphological and structural characteristics of the cement paste were studied.

The studies showed that it is most effective to use the PMA at the stage of binder preparing by grinding them together. In this case, an increase in compressive strength by 16.4% and tensile strength by 10% is observed. In this regard, the object of the presented manuscript was to study the component composition of the PMA and identify the role of each component in the modifying effect when synthesizing cement paste based on binders obtained by different additive introduction methods.

## 2. Materials and Methods

### Raw Materials

For the synthesis of PMA modified cement, PC clinker produced by Belgorod Cement CJSC (Belgorod city, Russia) was used. Its chemical composition is presented in Table 2. The assessment of its suitability for use in PMA-modified cement was carried out in accordance with Russian Standard GOST 31108-2020 [25] (Table 3).

Natural dihydrate gypsum (CaSO_4_·2H_2_O) from Novomoskovsk deposit (Russia) was used to regulate the binders’ setting process. Its chemical composition is presented in Table 4.

The possibility of using natural dihydrate gypsum in cement was assessed according to Russian Standard GOST 4013-2019 [26] (Table 5). PMA (Manufacturer LLC “Nickel”, Moscow, Russia) (Table 6), which is a granulated powder, was used as a functional component. Normally, it is used as a strengthening additive for slurries and dry mixes, to improve the physical and mechanical properties of crushed stone–gravel–sand mixtures, soils, and asphalt granule concrete mixtures used in structural layers of road pavement during construction, reconstruction, major repairs, and repair of highways [27,28,29].

According to patents [31,32] and open literature data, the components of PMA are the following: activated silica, redispersible lignin-based powder polymer, calcium, and sodium salts.

The binders (B1, B2, and B3) studied in the work were produced by grinding in a ball mill. B1 was obtained without PMA and contains only PC clinker—85% and gypsum—5%. B2 was obtained by dry mixing of B1 with 10% PMA. B3 or complex mineral binder was obtained as a result of combined grinding of PC clinker—85%, gypsum—5%, and PMA—10%.

The structural features of PMA were studied using an AXIO SCOPE A1 laboratory research microscope (Carl Zeiss, Oberkochen, Germany) and a TESCAN MIRA 3 LMU scanning electron microscope (TESCAN, Brno-Kohoutovice, Czech Republic). The elemental composition of individual zones in the PMA was determined using an Oxford Instruments NanoAnalysis X-MAX 50 energy dispersive spectrometer (Oxford Instruments, Oxfordshire, UK) based on an electron microscope.

The chemical composition of PMA and B1–B3 was determined using X-ray fluorescence (XRF) analysis, and the mineral composition was determined using X-ray diffraction (XRD) analysis using an ARL 9900 WorkStation series spectrometer with a built-in diffraction system (Thermo Fisher Scientific, Waltham, MA, USA). Diffraction patterns were recorded on a Co anode in the 2θ angle range from 8 to 80°. Qualitative analysis of the X-ray diffraction pattern with mineral identification was performed using the PDF-2 database of the International Center for Diffraction Data (ICCD). The qualitative composition of the binders was determined using infrared spectroscopy (IR) with a Vertex 70 FTIR spectrometer (Bruker Optics, Billerica, MA, USA). The test samples were pressed tablets of a studied mixture with potassium bromide. The absorption spectra in the range of 400–4000 cm^−1^ were obtained as the analyzed pattern. Also, to study the effect of PMA on the phase composition of hardened cement pastes, the methods of differential scanning calorimetry (DSC) and thermogravimetry (TGA) were used, implemented using a STA 449 F1 Jupiter device from NETZSCH (Selb, Germany).

## 3. Results and Discussions

### 3.1. Characteristics and Properties of PMA

**Figure 1:**
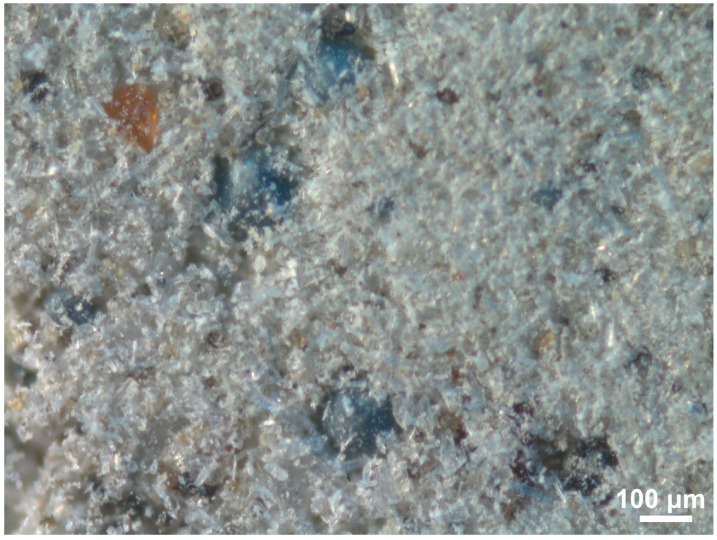
Macrostructure of PMA.

**Figure 2:**
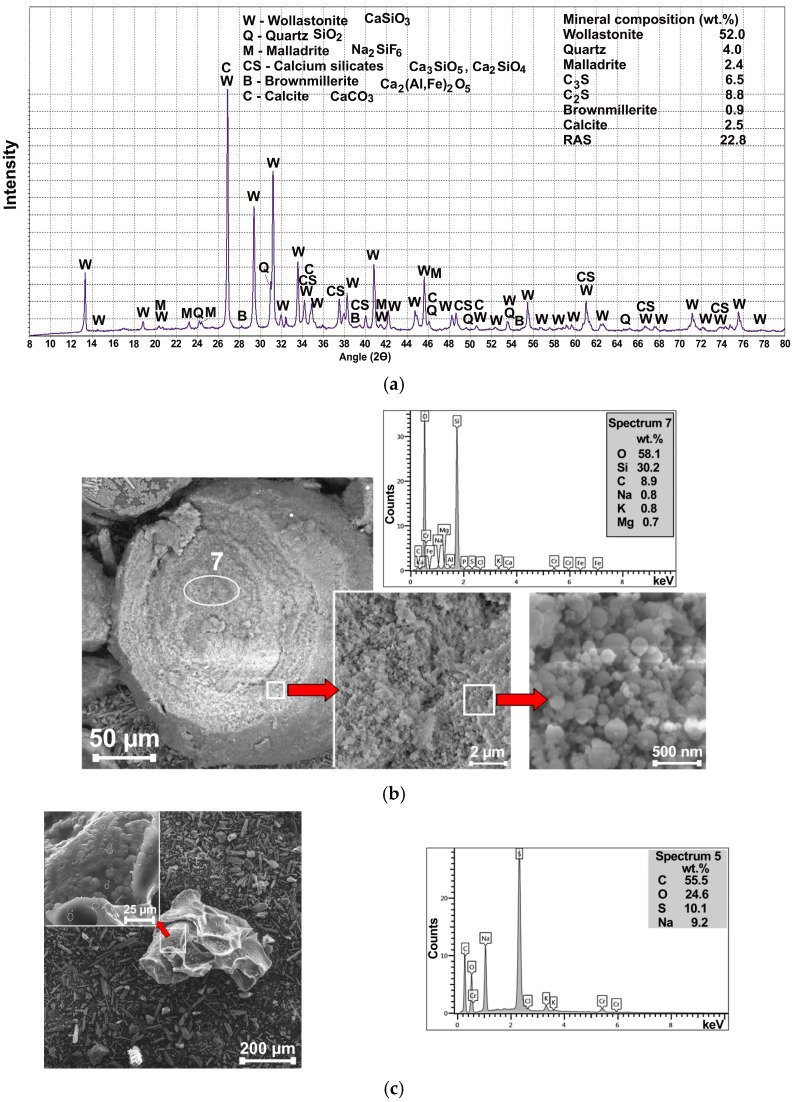
XRD, SEM, and EDS analysis of PMA: (**a**) XRD-profile of PMA; (**b**) SEM and EDS analysis of spherical granules in PMA; (**c**) SEM and EDS analysis of irregularly shaped particles in PMA.

PMA is a light-grey polydisperse powder containing dark-grey spherical granules up to 400 μm in size (Figure 1). Mostly, needle-shaped crystals predominate. In addition, there are orange-brown scale-shaped particles with a size of about 100 μm. The results of X-ray analysis show that the main crystalline phase is wollastonite Ca_3_Si_3_O_9_ (Figure 2a).

**Figure 3:**
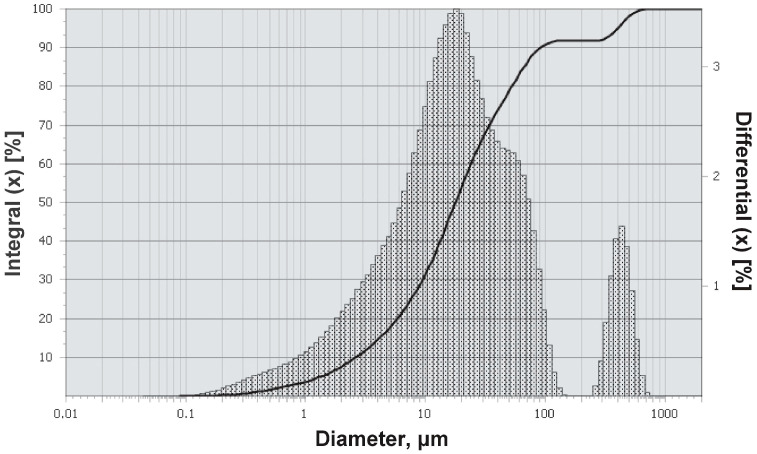
Granulometry of PMA.

SEM and EDS analysis revealed the following (Figure 2b,c): A significant proportion of the PMA is occupied by granules of predominantly spherical shape or their fragments (the size of an individual grain in the SEM image is about 250 μm) (Figure 2b). These particles have a porous structure and consist of small spheres of amorphous silica with a diameter of 50–100 nm. Based on the Si/O ratio (Si is 30.2 wt.%; O is 58.1 wt.%), the chemical formula of this substance is close to opal—SiO_2_·nH_2_O. Irregularly flake-shaped or crumpled-sheet-of-paper-shaped particles (Figure 2c) are presumably a condensation product of sodium salts of naphthalene sulfonic acid and formaldehyde, having the chemical formula C_10_H_7_SO_3_Na. This substance is a light-brown powder, acts as a naphthalene-based superplasticizer and is used to reduce the water demand in concrete mixtures [33]. The dispersion properties of PMA were assessed by several characteristics, such as measured specific surface (SSA) (according to the Blaine method), with SSA calculated according to data of particle size distribution (using laser granulometry) (Table 6, Figure 3). Based on Table 7, the SSA values obtained by different methods are comparable to each other.

According to the differential particle size distribution curve (Figure 3), PMA has three modes in the particle size range of 1–20 μm, 2–50 μm, and 3–400–500 μm. The integral distribution curve shows that more than 90% of the PMA volume is particles up to 100 μm. The presence of two peaks in this range is explained by the presence of particles of different mineral phases, as well as the differentiation of their shape. The third peak on the differential curve characterizes dark-gray granules.

The results of XRF analysis indicate the prevalence of silicon (SiO_2_ = 44.78%) and calcium (CaO = 43.08%) oxides in PMA (Table 8). There is a fairly high content of fluorine (F = 4.74%), which may indicate the presence of fluorides. The high proportion of loss on ignition in PMA is explained by the presence of organics and carbonates. According to the results of full-profile XRD analysis using Siroquant V3 software, it is evident that the main crystalline phase is wollastonite Ca_3_Si_3_O_9_, the content of which is 52.0% (Figure 2a). The presence of wollastonite (Ca_3_Si_3_O_9_ or CaSiO_3_ or CaO·SiO_2_) is consistent with the results of chemical analysis, where the SiO_2_/CaO ratio is 1.04, and the SiO_2_/CaO ratio is 1.07.

In addition to wollastonite, the following reflections of other minerals were detected: quartz, highly basic calcium silicates, calcite, brownmillerite, and malladrite (sodium hexafluorosilicate) (Figure 2a).

Despite the intensive background on the X-ray profile, the calculation using internal standards (anatase TiO_2_) showed a relatively small content of the X-ray amorphous phase (RAS) at 22.8%. It can include a polymer that is part PMA, as well as oxides that are not included in the recognized crystalline minerals, such as MgO, SO_3_, K_2_O, and others (Table 8).

**Figure 4:**
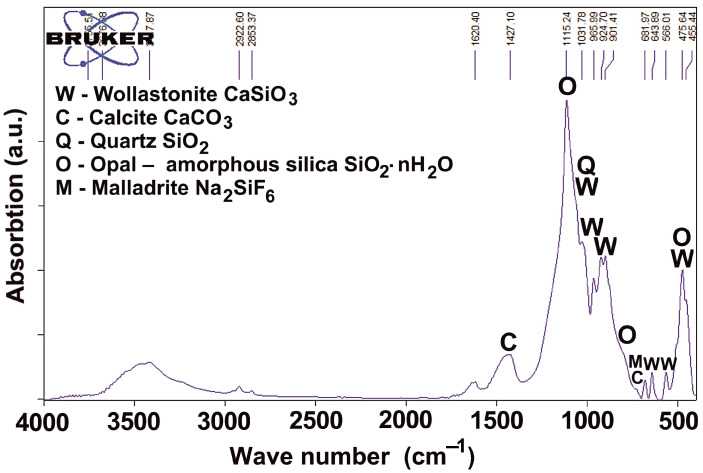
IR-spectra of PMA.

PMA was also studied by IR spectroscopy (Figure 4). The most intense absorption bands are located in the range of 700–1300 cm^−1^, which are characteristic of silicon–oxygen motifs in crystalline minerals and in the vitreous phase. The most intense absorption band at 1115 cm^−1^ characterizes opal SiO_2_·nH_2_O or amorphous silica. It is also characterized by bands at 800 and 470 cm^−1^, which are superimposed on the vibrations of bonds for other phases.

IR spectroscopy results show that the main mineral of PMA is wollastonite (absorption bands at 1090, 1060, 1032, 1020, 966, 925, 901, 682, 644, 566, 505, 476, 455 cm^−1^). Quartz profiles are superimposed on the absorption bands of amorphous silica and wollastonite (1171, 1084, 798, 779, 696, 513, 459 cm^−1^). Calcite (1427, 877, 712 cm^−1^) and malladrite (730 cm^−1^) are also detected.

**Figure 5:**
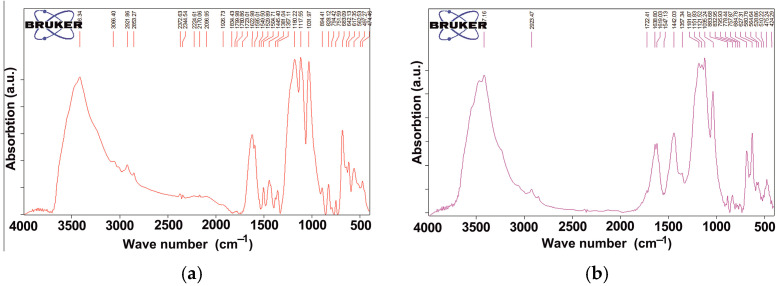
IR-spectra for (**a**) orange particles in PMA; (**b**) dried film of PMA after evaporation.

Since the polymer component of PMA is water-soluble, PMA was mixed with water and filtered through a paper filter. The aqueous extract was then evaporated, and the remaining film was studied by IR spectroscopy (Figure 5).

**Figure 6:**
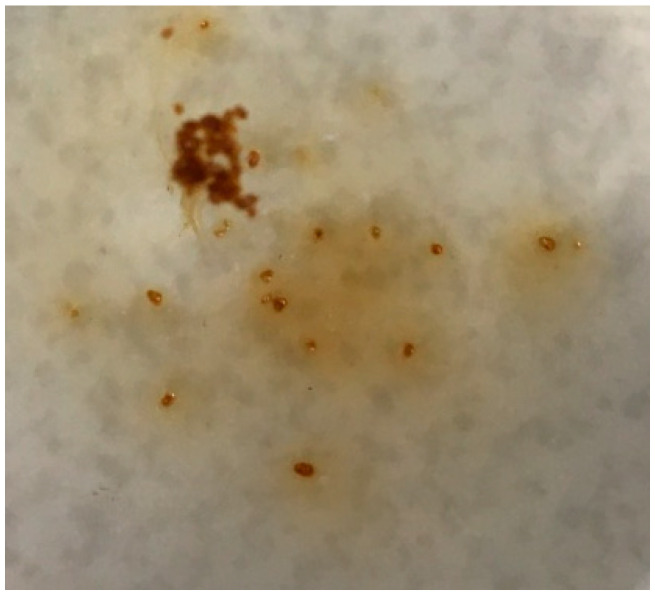
Dissolution of orange polymer particles in water.

The number of absorption bands detected in the spectrum indicates the presence of a water-soluble redispersible polymer in PMA (Figure 6).

The OPUS software (Version 7.2) database revealed the greatest number of matches in the obtained spectrum with the condensation product of sodium salts of naphthalenesulfonic acid. This compound is a surfactant used as a dispersing agent with the molecular formula of C_21_H_14_Na_2_O_6_S_2_.

SEM analysis revealed the elemental composition of the polymer component in PMA (Table 9), confirming the results of IR spectroscopy.

An analogue of the condensation product of sodium salts of naphthalenesulfonic acid is sodium naphthalenesulfonate or sodium naphthalene-2-sulfonate (C_10_H_7_SO_3_Na), which is a superplasticizer substance based on naphthalene and is used to reduce the water demand in concrete mix. It is a light-brown powder, soluble in water, with a pH of 2.5%, whose aqueous solution is 7–9. These data are in good agreement with the obtained results.

Thus, PMA is a mixture of various organic and mineral substances that participate in the structure formation of cement paste during hydration. Studies carried out allowed us to clarify the components of PMA and establish their possible influence on the properties of cement.

### 3.2. Characteristics of PMA-Modified Binders

**Figure 7:**
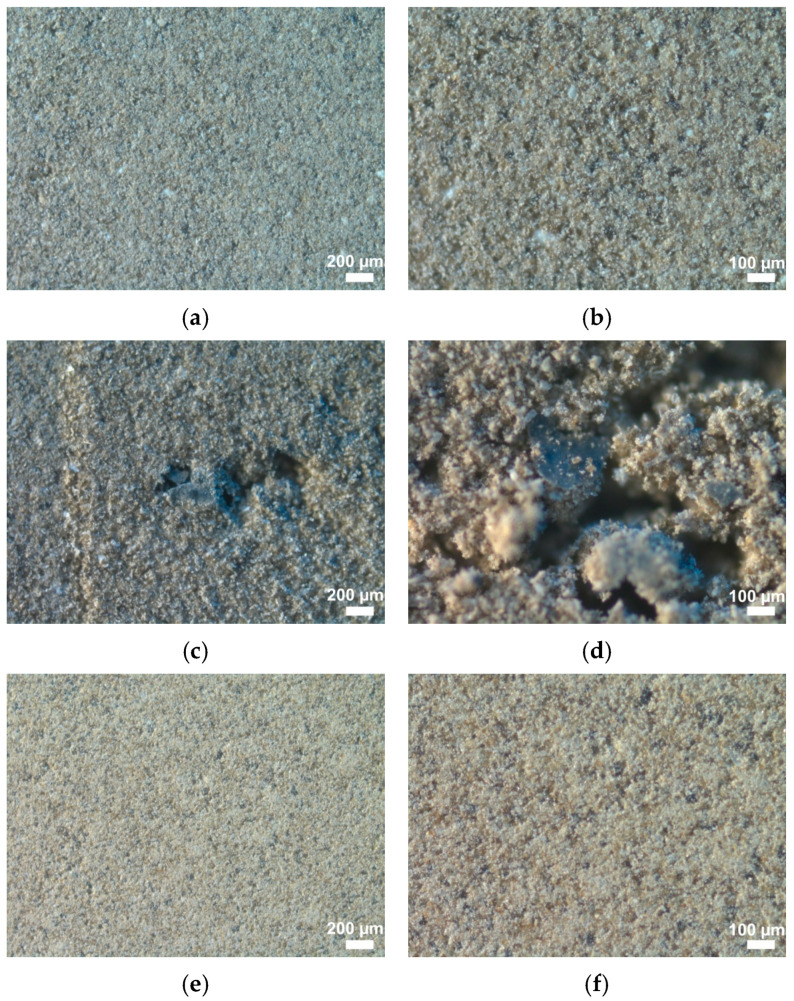
Microstructure of the studied binders: (**a**,**b**) B1; (**c**,**d**) B2; (**e**,**f**) B3.

The mechanism of PMA-modified binder (B1, B2, B3) hardening and the formation of the final structure, which largely determines the properties of the final composite, primarily depends on the distribution uniformity degree of the components in the binder volume. This parameter is easiest to evaluate visually based on optical microscope images (Figure 7).

The study of the chemical composition of binders B1–B3 made it possible to identify the effects of PMA (Table 10).

**Figure 8:**
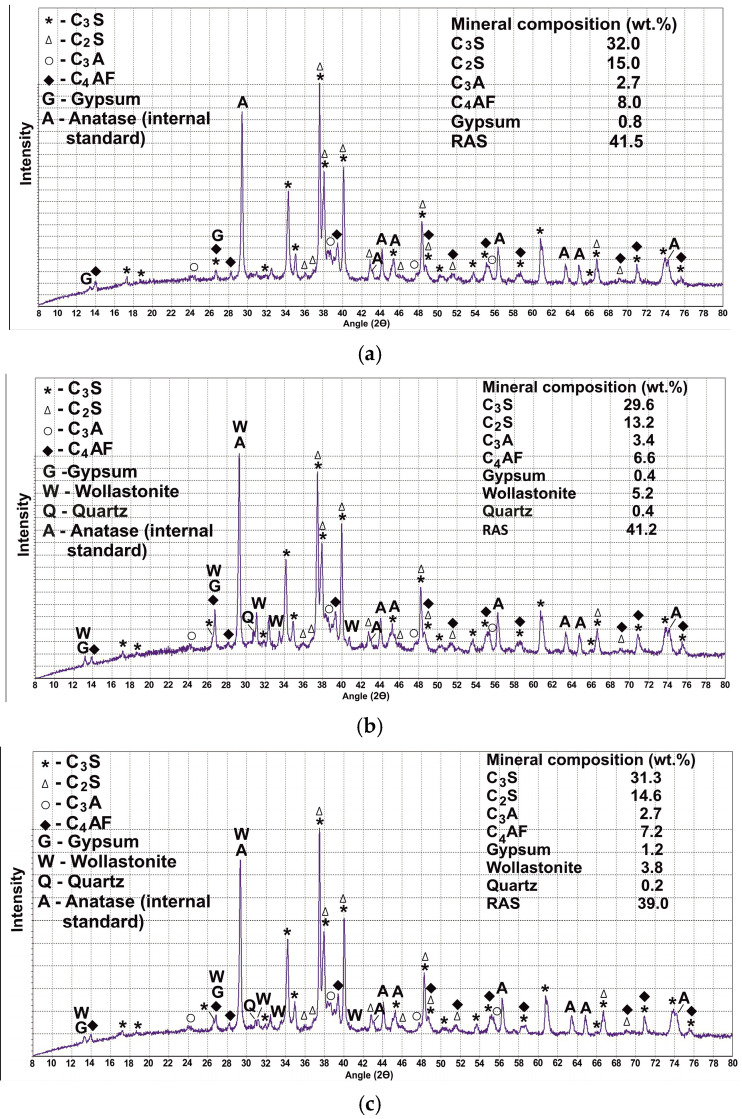
X-ray profiles of (**a**) B1; (**b**) B2; (**c**) B3.

B1 meets the requirements of Russian Standard GOST 31108-2020 in chemical composition; MgO is 3.03% (according to GOST no more than 5%), SO_3_ is 2.45% (according to GOST no more than 3.5%), and chloride ions were not found (according to GOST no more than 0.1%). The composition of the synthesized binder B1 is close to the materials from other studies [34,35,36,37]. The addition of 10% PMA (by wt.% of the total binder) was chosen as optimal [27] and affects the content of the main oxides of the cement-based binder; CaO, Al_2_O_3_, Fe_2_O_3_, and MgO decrease, and the content of SiO_2_ increases (Table 10). However, the addition of PMA and, as a consequence, the change in the chemical composition of the cement binder do not affect its compliance with the requirements of GOST 31108-2020. X-ray profiles for PMA-bearing binders, regardless of the introduction method (dry mixing or combined grinding, B2 and B2, respectively) demonstrate reflections of the main crystalline phases of the PMA, identified as wollastonite (5.2% and 3.8% of the crystalline part of the binders, respectively), and, to a much lesser extent, quartz (0.4% and 0.2% of the crystalline part of the binders, respectively) (Figure 8).

**Figure 9:**
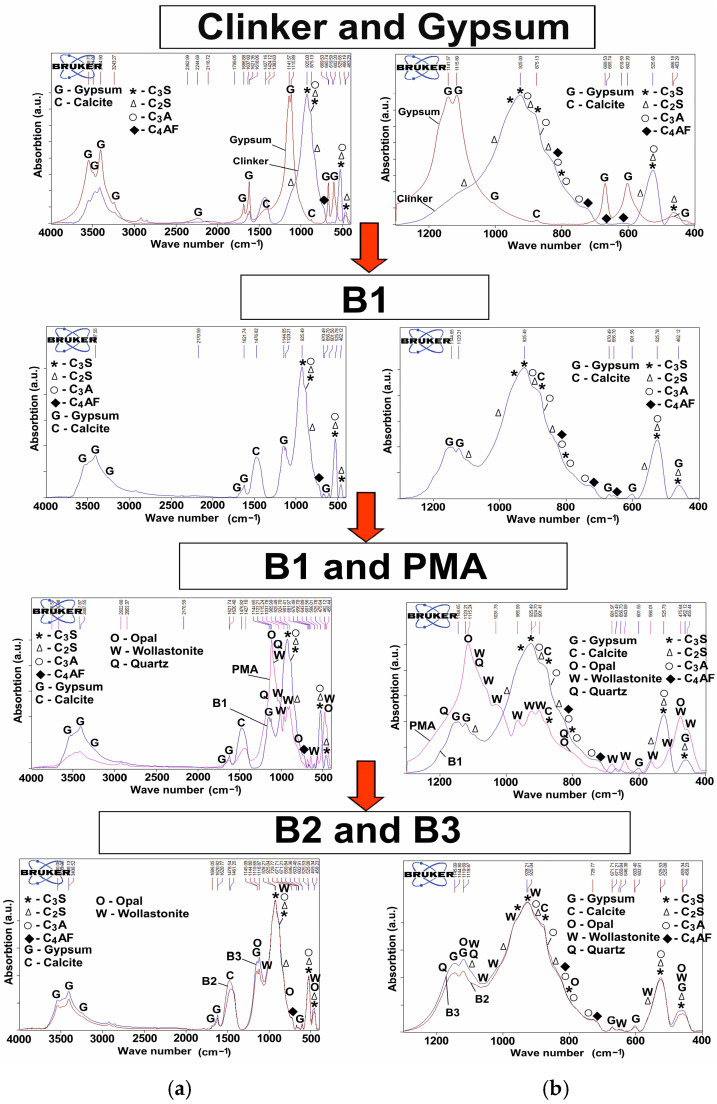
IR-spectra for the binders in different ranges of wave numbers: (**a**) 500–4000 cm^−1^; (**b**) 400–1200 cm^−1^.

Quantitative XRD analysis using an internal standard (anatase TiO_2_) showed a fairly high content of the RAS in the synthesized B1 at 41.5%. When replacing 1/10 of the PC with PMA with a lower content of RAS (22.8 wt.%), the amount of the RAS in binders B2 and B3 decreases slightly. The lowest content of RAS is noted for B3 at 39% (Figure 8). The results of IR spectroscopy showed that the IR spectrum for B1 is the sum of the IR spectra of PC clinker and gypsum (Figure 9). Clinker is characterized by the presence of a wide absorption band in the range of 700–1200 cm^−1^, with a large number of obscure peaks that can be attributed to the absorption bands of clinker minerals [38,39,40]. Gypsum appears to be a fairly pure substance (absorption bands at 3549, 3495, 3405, 3243, 2245, 2117, 1687, 1621, 1142, 1116, 1007, 670, 602, 466 cm^−1^). Calcite is present as an impurity (1424, 875 cm^−1^), which was also identified according to XRD analysis (its content in natural gypsum is 5.1 wt.%). Gypsum peaks are well identified in the spectrum for all studied binders, in the ranges of 3000–4000 cm^−1^, 1100–1200 cm^−1^, and 600–700 cm^−1^. The addition of PMA does not practically change the profile of B1 (Figure 9). This is due to the fact that the main peaks of PMA overlap with the absorption bands of PC clinker and gypsum. The difference lies in the increase in the spectral line in the absorption bands typical of PMA, namely at 1000–1200 cm^−1^ and 400–500 cm^−1^, and the appearance of some obscure peaks characteristic primarily of wollastonite in PMA.

The higher intensity of the absorption bands at 1145, 1120, and 459 cm^−1^ in the IR profile for B3 compared to B2 can be explained by a more uniform distribution of amorphous silica in the binder, since its key absorption bands are located in these spectrum ranges (Figure 9).

The results obtained will be further used to establish the relationship between the method of PMA introduction and the structure formation of the cement matrix based on three different binders. It is well known that the composition, quantity, and distribution of newly formed hydrates in cement paste have a key effect on the microstructural characteristics (total porosity, pore size distribution, presence of microcracks, etc.) and durability of concrete or other composite material. The main hydrate phases are portlandite Ca(OH)_2_, calcium hydrosilicates C-S-H, and calcium hydrosulfoaluminates (ettringite and monosulfoaluminate). Cement paste can be considered as a composite material, where calcium hydroxide and calcium hydrosulfoaluminate crystals, as well as unreacted PC particles, are embedded in a matrix of C-S-H gel [41,42,43]. This gel is considered the most important hydration product, since it is the main cementitious phase of the PC-based system. Therefore, it is the calcium silicate hydrates C-S-H that make a significant contribution to the mechanical (primarily strength) properties of cement paste.

**Figure 10:**
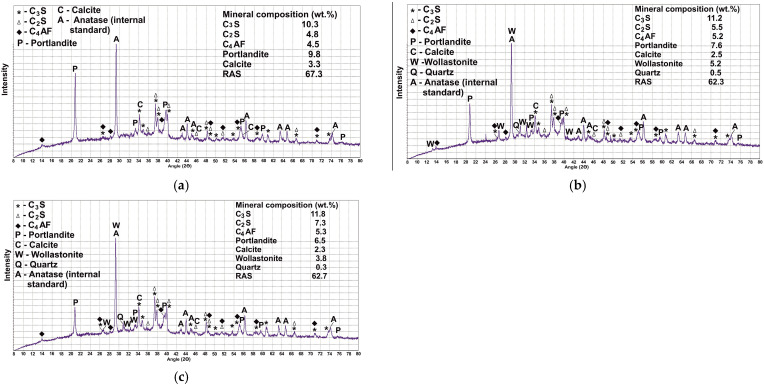
XRD-profile of the 28-days binders: (**a**) B1; (**b**) B2; (**c**) B3.

To characterize the structure and composition of cement paste, several analytical methods are usually used. The X-ray diffraction method allows identifying the following crystalline phases: PC clinker minerals (alite, belite, and brownmillerite), portlandite Ca(OH)_2_, and calcite CaCO_3_ as a product of calcium hydroxide carbonation (Figure 10a). They were diagnosed in a 28-day sample of cement paste based on B1 (CP-B1). Also, based on calculations using an internal standard (anatase TiO_2_), the content of RAS was determined to be 67.3% (Figure 10a). As expected, cement hydration leads to a significant increase in the RAS content (by almost 26%) (Table 11). This is confirmed by the results of other studies [44,45]. At the same time, the amount of all clinker phases decreases. The greatest decrease is observed for alite.

Tricalcium aluminate is not identified on the X-ray profile. The content of portlandite in the cement paste based on B2 (CP-B2) and cement paste based on B3 (CP-B3) decreases (Figure 10, Table 11). It is the least for CP-B3. A similar trend is characteristic of calcite. Noteworthy is a slight decrease in RAS, compared to B1 (62.3% and 62.7% for CP-B2 and CP-B3, respectively). This can be explained by its initially lower content in the binders, as well as a lower amount of PC (by 10%). During hydration the greatest changes in clinker phases occur, and the PMA content (its mineral crystalline part) remains unchanged. Therefore, the main crystalline phases of PMA (wollastonite and quartz) can be used as a certain internal standard. In this work, based on the calculation results, the equality of the amount of these phases in the binder before and after hydration (taking into account RAS) was established (Table 11).

**Figure 11:**
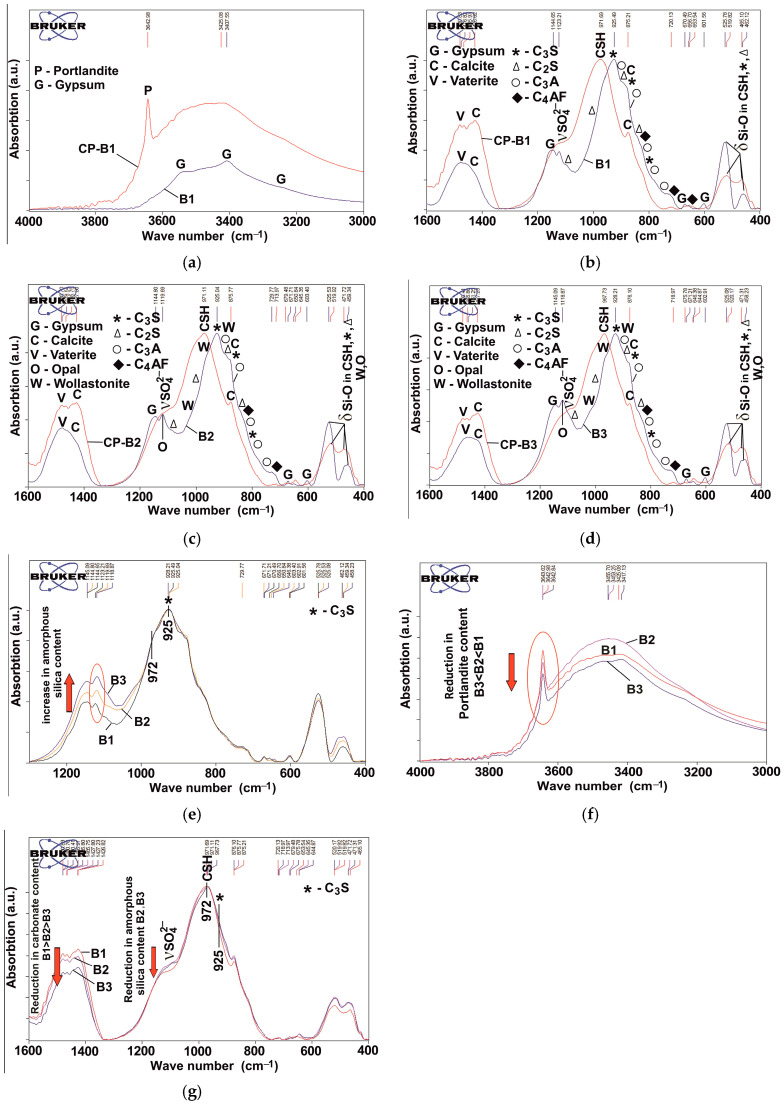
IR spectra for the binders and hydrated cement paste (CP): (**a**) B1 and CP-B1 in range of 3000–4000 cm^−1^; (**b**) B1 and CP-B1 in range of 400–1600 cm^−1^; (**c**) B2 and CP-B2 in range of 400–1600 cm^−1^; (**d**) B3 and CP-B3 in range of 400–1600 cm^−1^; CP based on B1, B2, and B3 at different ranges: (**e**) 400–1300 cm^−1^; (**f**) 3000–4000 cm^−1^; (**g**) 400–1600 cm^−1^.

The reduced content of RAS in CP-B2 and CP-B3 can indicate a lower amount of C-S-H gel. However, this does not affect the strength characteristics of the final material, since these binders initially include wollastonite, a mineral consisting of chains of diorthogroups [Si_2_O_7_], which are elements of the C-S-H gel structure. When structuring the cement matrix, it complements the newly formed calcium hydrosilicate formations without participating in chemical reactions, which shows the absence of changes in the calculated amount of wollastonite [46]. It is also worth noting that the smaller the size of individual particles of the anisotropic mineral, the closer they are to hydrated formations in size. Therefore, the use of B3 is more effective than B2. In addition, the best results of physical and mechanical properties are ensured by the uniform distribution of the PMA components. Since the C-S-H gel phase in the cement paste is X-ray amorphous, it is not possible to identify it by X-ray diffraction, which is why IR spectroscopy was used in the work. IR spectroscopy studies (Figure 11) showed that as a result of PC hydration, its main peak at 925 cm^−1^, which characterizes the antisymmetric stretching vibrations of Si-O in the main phase of clinker alite, shifts to the area of higher wave numbers up to 972 cm^−1^. This indicates the polymerization of silicate groups [SiO_4_]^4−^ and the formation of C-S-H gel [47] (Figure 11b). Based on the position of this band, researchers characterize its belonging to chain silicates (Q^2^), which are calcium hydrosilicates of the jennite or C-S-H(II) type [48]. They are characterized by a high CaO/SiO_2_ ratio (>1.2) and a solution pH of more than 11. A change in the structure of silicates in a significant proportion of the material is evidenced by a change in the profiles of deformation vibrations characterizing the change in angles for the Si-O-Si bonds: the absorption band of 462 cm^−1^ (oscillations in the plane) increases its intensity, and the absorption band of 526 cm^−1^ (oscillations out of the plane) shifts to 520 cm^−1^ and significantly loses its intensity.

Since the C-S-H gel is a hydrated phase, it is characterized by the presence of bound water, which explains the significant increase in the profile of stretching vibrations of O-H bonds (3000–3800 cm^−1^) (Figure 11a). In the same range, there is a sharp absorption band at 3643 cm^−1^, describing the stretching vibrations of O-H groups of the portlandite structure [49]. An inevitable consequence of hydration is carbonation, which is expressed in the IR spectra by a significant increase in the intensity of the calcium carbonate peaks in the ranges of 1400–1500 cm^−1^ and at 875 cm^−1^ (Figure 11b). The wide band at 1400–1500 cm^−1^ is divided into several separate bands. One of them with a maximum at 1427 cm^−1^ can be attributed to calcite; the second group of peaks in the region of 1465–1485 cm^−1^ is associated with vaterite. The appearance of vaterite is associated with carbonation of the C-S-H gel. It appears as cryptocrystalline new formations, which explains its absence in the X-ray profiles. During carbonation of portlandite, more crystallized calcite is formed.

The presence of a “shoulder” at 1100–1200 cm^−1^ to the main profile of the IR spectrum characterizes the presence of sulfate phases: calcium hydrosulfoaluminates (Figure 11b).

Hydration of B2 and B3 occurs in a similar way as for B1 (Figure 11c,d).

Small differences in the intensity of absorption bands in the ranges of 1100–1200 cm^−1^ and 400–600 cm^−1^ are associated with differences in the composition of the binders, caused by the introduction methods of PMA, as well as the speed and completeness of the hydration processes of clinker minerals.

Differences in the studied binders can be seen in the “fingerprint region” of the IR spectra (Figure 11d,f,g). As noted earlier, the increase in the intensity of the absorption bands at 1145, 1120, and 459 cm^−1^ for B2 and B3 is due to amorphous silica in PMA. At the same time, the increase in the height of the peaks in the range of 1100–1200 cm^−1^ (the spectra are normalized to the absorption band at ~925 cm^−1^, characteristic of alite) in the following sequence, CP-B1 → CP-B2 → CP-B3, can be explained by an increase in the content of amorphous silica due to its more uniform distribution in the binder. A slight decrease in the intensity of the peak at 525 cm^−1^ is due to a decrease in the content of the clinker component. Comparison of the IR spectra of the cement pastes based on different binders revealed some differences (Figure 11e,g). First of all, in CP-B2 and CP-B3, a relative increase in the unhydrated clinker component is observed relative to newly formed calcium hydrosilicates (the spectra are normalized by the absorption band at ~970 cm^−1^, characteristic of C-S-H gel). This can be judged by the increase in the profile of the main absorption band of clinker (mainly silicon–oxygen tetrahedrons) in the range of 800–~950 cm^−1^ (visible by the alite band at 925 cm^−1^) and the peak height at 520 cm^−1^ (Figure 11g). At the same time, in the binders before hydration, the relative heights of the peaks at 925 and 972 cm^−1^ completely coincide (Figure 11d). However, the decrease in the hydration degree does not lead to a significant decrease in the amount of C-S-H gel. The presence of amorphous silica in B2 and B3 determines the occurrence of pozzolanic reactions, leading to the formation of an additional amount of calcium hydrosilicates. This can be judged based on a significant decrease in the profile in the range of 1100–1200 cm^−1^ (Figure 11g), the intensities of which are very different from those of unhydrated binders (Figure 11d). The occurrence of pozzolanic reactions is also evidenced by a decrease in the height of the peak characteristic of portlandite (Figure 11e). It is worth noting that the number of reactions taking place and the involved volume of the substance is significantly higher when using B3 compared to B2. Changes in the intensities of the absorption bands of calcium hydroxide and amorphous silica are comparable. In addition, the increase in the profile intensity in the range of ~800–950 cm^−1^ can be explained by the presence of wollastonite, the main absorption bands of which lie precisely in this range.

The amount of formed calcium carbonates of different polymorphic modifications was judged by the absorption bands of the carbonate ion in the range of 1400–1500 cm^−1^ (Figure 11g). The sequence of changes in the carbonate content is consistent with the corresponding portlandite content as follows: CP-B3 → CP-B2 → CP-B1. All the obtained results are consistent with the XRD data.

Thus, joint grinding allows for uniform distribution of all components of the complex mineral binder mixture (clinker mineral phases, calcium sulfate, PMA components) by volume and their involvement in structure formation. At the same time, using the traditional mixing method, PMA particles are distributed unevenly, which significantly affects the quality of reactions as a result of binder hydration.

**Figure 12:**
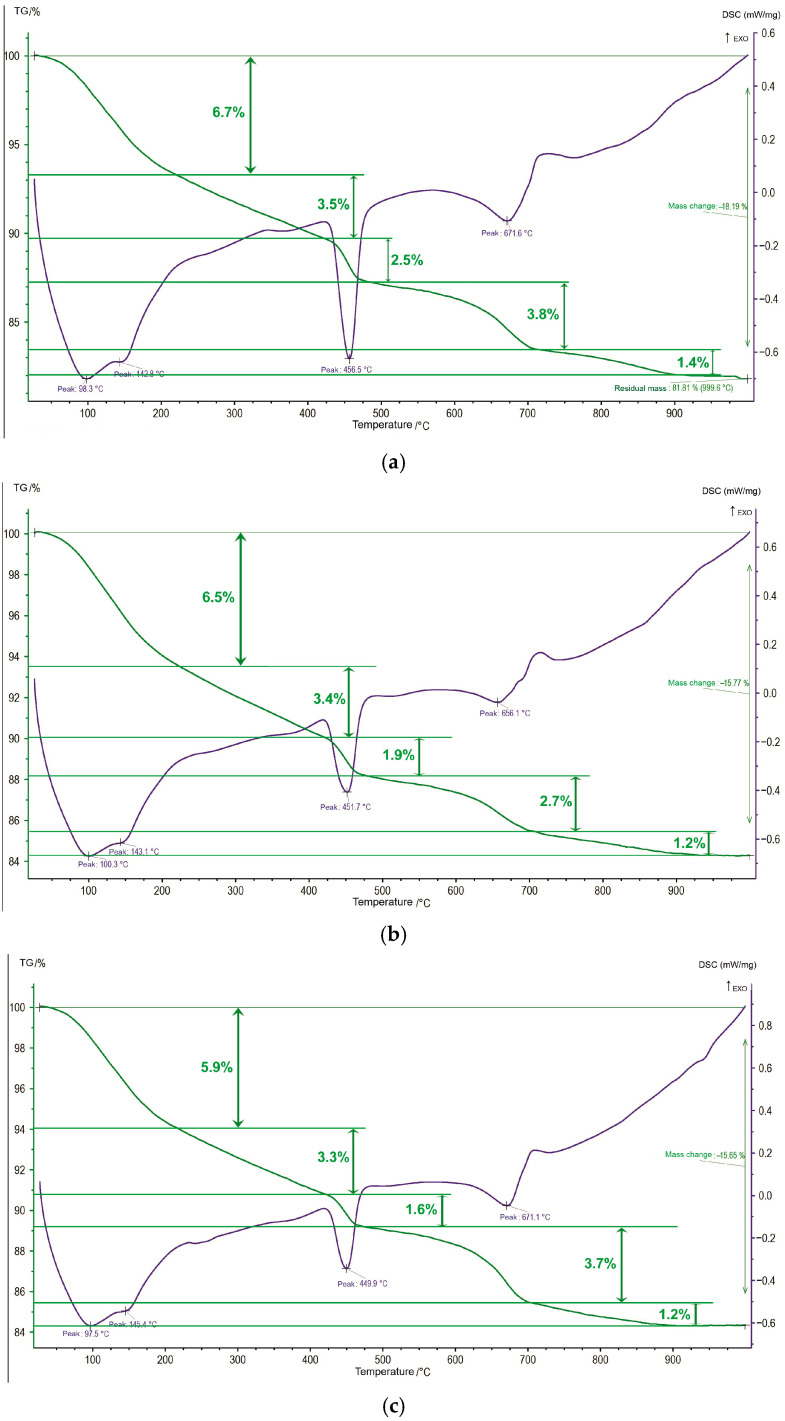
DTA for 28-day cement pastes based on different binders: (**a**) CP-B1; (**b**) CP-B2; (**c**) CP-B3.

In the classical interpretation of thermal analysis of cement paste, the following endothermic peaks are observed, which were found in the studied binders (Figure 12, Table 12):

(1) at 50–220 °C, a release of physically bound water from the pores and dehydration due to the loss of water from the layers of C-S-H gel and calcium hydrosulfoaluminates;
(2) at 220–420 °C, continuous thermal decomposition of a complex mixture of hydrated compounds of the silicate and aluminate type, such as C_4_AH_13_ (and, possibly, C_3_AH_6_);
(3) at 420–475 °C, dehydration and decomposition of portlandite;
(4) at 475–~710 °C, a loss of structural OH groups of C-S-H and decomposition of vaterite;
(5) at ~710–~900 °C, decomposition of calcite.

In the first region, the two following endothermic peaks are found: the first one in the range of 98–100 °C is associated with the loss of adsorbed water; the second one, at 143–145 °C, is explained by the removal of interlayer water from the C-S-H gel and the dehydration of calcium hydrosulfoaluminates. Carbonation of the C-S-H gel leads to the formation of vaterite, which is an unstable type of calcium carbonate. Therefore, it decomposes at a lower temperature than calcite. The temperature range of the corresponding decompositions is difficult to determine, since the peaks overlap. The decomposition temperature of vaterite for different binders is in the range of 656–672 °C. The decomposition reactions of calcium carbonates are accompanied by a significant loss of mass, which ranges from 3.9 to 5.2% depending on the binder (Table 12). Thermogravimetric analysis (TGA) is a convenient method for the quantitative determination of calcium carbonate and portlandite content.

The portlandite content was calculated with the results of TGA, using the known value of mass loss during decomposition of pure Ca(OH)_2_ (which is 24.34%) and the change in mass in the same temperature range of the studied cement pastes. The amount of carbonates was calculated in a similar way, based on the known value of mass loss during decomposition of pure CaCO_3_ (44%). However, the exact amount of carbonated phases, primarily vaterite, cannot be reliably calculated based on the mass loss data, since the structural OH groups in C-S-H gel also contribute to the measured mass loss in this temperature range. The calculated results are presented in Table 12.

**Figure 13:**
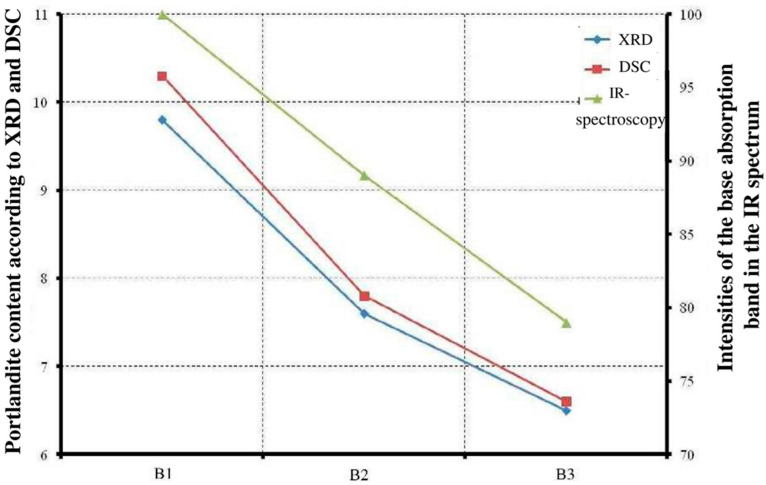
Dependence of portlandite content in cement pastes based on the studied binders.

The quantitative values of the portlandite content in 28-day cement pastes obtained by different methods are in good agreement with each other and are built into a certain dependence on the binder used. Portlandite is found most in B1, significantly less in B2, and least of all in B3 (Table 13, Figure 13).

This can be explained by the occurrence of pozzolanic reactions between calcium hydroxide and amorphous silica in PMA, which leads to the formation of an additional amount of calcium hydrosilicates. The intensity and completeness of these reactions are higher when using jointly grinded binder components. A decrease in free calcium hydroxide content is also confirmed by a general decrease in the carbonated phases’ amount (Table 13).

Carbonated phases’ content is more in PC-B1. The trend is quite clearly visible in the example of calcite, although it differs slightly from the dependence for portlandite. It is more difficult to judge for vaterite, since, being X-ray amorphous, the mass loss taken from TGA also includes the loss of structural OH groups of the C-S-H gel.

As can be seen from Table 13, the calculated values of portlandite and calcite according to XRD and TGA data are quite close, which confirms the reliability of the results obtained and the calculations performed.

Also, using the obtained value of the portlandite content from the TGA data and accepting its full crystallinity, the amount of RAS was calculated (Table 14). The obtained values of RAS were close to the XRD calculation results using an internal standard with the preservation of the following dependence between the compositions: CP-B1 > CP-B3 > CP-B2. This method is not new in the practice of cement researchers.

**Figure 14:**
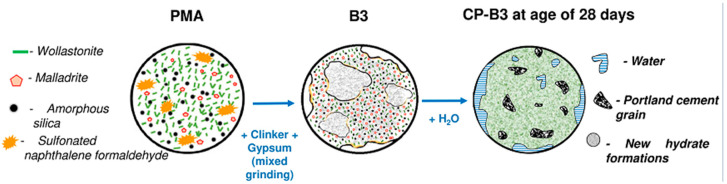
Phenomenological model of structure formation for CP-B3.

Based on the data of the component composition of PMA, a phenomenological model of the CP-B3 structure formation (B3 is obtained by joint grinding of the components of the “cement clinker—gypsum—polymer–mineral additive”) is proposed (Figure 14).

The plasticizing component of PMA ensures a decrease in water demand. Grinding of amorphous silica granules promotes an increase in its reactivity and an increase in the surface of interaction with calcium hydroxide during pozzolanic reactions. Wollastonite particles create additional micro-reinforcement and act as a substrate for the formation of hydration product nuclei and their growth. Homogenization of the binder components leads to a uniform filling of the hardening matrix with additional new formations. As a result, a more compact condensation-crystallization structure is formed, providing improved physical and mechanical properties.

## 4. Conclusions

Thus, each component of PMA has a different effect on the properties of the PC, and this effect is most clearly manifested in the production of a composite binder, when mechanical and chemical activation not only of the added PMA occurs, but also of the entire mixture as a whole. In addition, when using a complex polyphase polymer–mineral composition, a synergistic effect of the PMA can be realized, enhancing the action of each of them, as evidenced by the data obtained by other researchers [50]. Based on the implemented studies, the interaction of PMA components with a PC as a result of grinding and subsequent hydration of a complex mineral binder can be represented in the form of the following phenomenological model: grinding of amorphous silica granules promotes an increase in its chemical activity and an increase in the surface area of interaction with calcium hydroxide during pozzolanic reactions; wollastonite particles create additional micro-reinforcement and act as a substrate for the formation of hydration product nuclei and their growth; homogenization of the system leads to uniform filling of the hardening matrix with additional new formations. As a result, a more compact condensation-crystallization structure is formed, providing increased physical and mechanical properties of the cement paste.

Thus, based on the data obtained with a wide range of analytical methods and high-precision equipment, the composition of PMA was analyzed; the mechanism of its interaction with the hydration products of the PC was established depending on the PMA introduction method. Obviously, joint grinding of PC with PMA initiates the intensity of the hydration reactions of the resulting composite binder, which is higher, which is also proven by the obtained earlier results [23] of physical and mechanical tests of PMA-bearing binders.

Despite the proven increase in the efficiency of the composite mineral binder in this manuscript, it is advisable to study the effect of the binder and the polyfunctional additive on the physical, mechanical, and operational properties of multi-component systems for various areas of construction. This will allow us to identify the most rational areas of application of the complex mineral binder containing the polymer–mineral additive, to design optimal compositions of composites using it, and to predict the operational reliability and durability of materials based on this binder.

Also, based on the totality of the obtained data, taking into account the possibility of partial replacement of the clinker component in a complex mineral binder with technogenic raw materials, in further studies it is advisable to consider the efficiency of using such large-tonnage waste as fly ash and metallurgical slag in PMAs. Taking into account the multi-functional purpose of both PMAs and complex mineral binders with their use, it is of great interest to study the influence of the binder on the physical, mechanical, and water–physical properties of clay systems with various compositions to use in road construction.

## Figures and Tables

**Table 1:** Physical and mechanical properties of the cement paste according to [24].

Binder ID	Standard Consistency, %	Initial Setting Time, min	28-Day Strength, MPa	
			Compressive	Tensile
B1	28	133	29.4	5.4
B2	27	128	33.1	5.6
B3	27.5	123	35.2	6.0

**Table 2:** Chemical composition of PC clinker.

Oxides Content, %									
CaO	SiO_2_	Al_2_O_3_	Fe_2_O_3_	MgO	K_2_O	Na_2_O	TiO_2_	SO_3_	Others
64.83	20.44	5.72	4.51	1.81	1.16	0.38	0.33	0.28	0.54

**Table 3:** The content of basic standardized oxides and components of PC clinker according to Russian Standard GOST 31108-2020.

Parameter	According to GOST 31108-2020	Real
Total content of tricalcium and dicalcium silicates (3CaO SiO_2_ + 2CaO SiO_2_)	no more than 2/3 of the clinker weight	74.25%
Calcium oxide to silicon oxide ratio (CaO/SiO_2_), by wt.%	no more than 2.0	3.17
Magnesium oxide (MgO) content, by wt.%	no more than 5.0 of the clinker weight	1.81

**Table 4:** Chemical composition of natural dihydrate gypsum (CaSO_4_·2H_2_O).

Oxides Content, %					
CaO	SO_3_	SrO_2_	MgO	Others	LOI
32.34	45.97	0.23	0.16	0.15	21.05

**Table 5:** Content of gypsum and crystallization water in natural dihydrate gypsum.

Component	Content According to GOST 4013-2019	Real Content	Type According to GOST 4013-2019
Natural dihydrate gypsum (CaSO_4_·2H_2_O)	not less than 70	99.4	1
Crystallization water	not less than 14.6	20.8	

**Table 6:** Chemical composition of PMA according to Organization standard 13881083.002-2021 [30].

CaO	SiO_2_	Al_2_O_3_	Fe_2_O_3_	MgO	F	Others
45.0 ± 5.0	43.0 ± 5.0	1.1 ± 0.3	1.4 ± 0.3	1.1 ± 0.4	4.4 ± 1.0	4.0 ± 1.0

**Table 7:** SSA data of PMA.

Parameter ID	Measured (by Blaine Method)	Calculated (by Laser Granulometry)
SSA, m^2^/kg	479.1	416.9

**Table 8:** Chemical composition of PMA.

Oxides Content, %											
SiO_2_	CaO	F	Al_2_O_3_	MgO	Na_2_O	SO_3_	Fe_2_O_3_	K_2_O	P_2_O_5_	Others	LOI
44.78	43.08	4.74	1.49	1.45	1.39	1.25	1.12	0.30	0.09	0.31	11.2

**Table 9:** Elemental composition of water-soluble redispersible polymer in PMA.

Component		Element Content, wt.% *			
		C	O	S	Na
Polymer	Spectrum 4	54.9	23.8	10.9	9.4
	Spectrum 5	55.5	24.6	10.1	9.2
Condensation product of sodium salts of naphthalenesulfonic acid (C_21_H_14_Na_2_O_6_S_2_)		55.0	21.0	14.0	10.0

* Electron microscope-based EDS spectrometer does not detect hydrogen (H).

**Table 10:** Chemical compositions of the studied binders.

Binder ID	Oxides Content, wt.%										
	CaO	SiO_2_	Al_2_O_3_	Fe_2_O_3_	MgO	SO_3_	K_2_O	Na_2_O	TiO_2_	F	Others
B1	61.62	20.60	6.32	3.95	3.03	2.45	0.78	0.44	0.23	-	0.58
B2	59.71	23.58	5.83	3.64	2.90	2.32	0.73	0.57	0.21	-	0.51
B3	59.33	23.39	5.52	3.56	2.52	2.69	0.77	0.88	0.21	0.62	0.51

**Table 11:** Variations in the mineral composition of the binders at 28-days of age.

Mineral Phase	Content, wt.%					
	B1		B2		B3	
	Without Taking into Account RAS	Taking into Account RAS	Without Taking into Account RAS	Taking into Account RAS	Without Taking into Account RAS	Taking into Account RAS
Ca(OH)_2_	+30.0	+9.8	+20.1	+7.6	+17.4	+6.5
C_3_S	−23.3	−21.7	−20.7	−18.4	−19.7	−19.5
C_2_S	−11.0	−10.2	−7.8	−7.7	−4.4	−7.3
C_3_A	−4.6	−2.7	−5.7	−3.4	−4.4	−2.7
C_4_AF	+0.2	−3.5	+2.6	−1.4	+2.4	−1.9
Gypsum	−1.3	−0.8	−0.6	−0.4	−1.9	−1.2
CaCO_3_	+10.0	+3.3	+6.6	+2.5	+6.2	+2.3
Wollastonite	-	-	+4.9	0.0	+4.0	0.0
Quartz	-	-	+0.6	+0.1	+0.4	+0.1
RAS	-	+25.8	-	+21.1	-	+23.7

**Table 12:** Change in the mass of 28-day cement paste using the studied binders in different temperature ranges and the calculated content of individual phases.

Temperature Range, °C	Chemical Process	B1		B2		B3	
		Weight Loss	Phase Content	Weight Loss	Phase Content	Weight Loss	Phase Content
25–220	Removal of water and dehydration of the C-S-H gel and calcium hydrosulfoaluminates	6.7	-	6.5	-	5.9	-
220–420	Dehydration of the C_4_AH_13_ phase (and possibly the C_3_AH_6_ phase)	3.5	-	3.4	-	3.3	-
420–475	Portlandite dehydration	2.5	10.3	1.9	7.8	1.6	6.6
475–~710	Loss of structural OH groups in C-S-H and vaterite decomposition	3.8	8.61	2.7	6.11	3.7	8.4 *
~710–~900	Calcite decomposition	1.4	3.2	1.2	2.7	1.2	2.7

* phase content calculated for vaterite CaCO_3_.

**Table 13:** Contents of portlandite and carbonate phases in cement paste using the studied binders, determined by various methods.

Binder ID	Mineral Phase	Research Method	
		XRD	TGA
B1	portlandite	9.8	10.3
	calcite	3.3	3.2
	vaterite	-	8.6
B2	portlandite	7.6	7.8
	calcite	2.5	2.7
	vaterite	-	6.1
B3	portlandite	6.5	6.6
	calcite	2.3	2.7
	vaterite	-	8.4

**Table 14:** RAS amount in the studied cement pastes calculated by different methods.

Binder ID	XRD	XRD + TGA
B1	67.3	65.7
B2	62.3	61.2
B3	62.7	62.1

## Data Availability

The original contributions presented in the study are included in the article, further inquiries can be directed to the corresponding author.
