# Correction: Strokova et al. Interaction Mechanisms in «Portland Cement—Functional Polymer Mineral Additives» Binder Produced by Different Methods. *Materials* 2025, *18*, 3178

**DOI:** 10.3390/ma18184373

**Published:** 2025-09-19

**Authors:** Svetlana Bondarenko, Mikhail Lebedev, Valeria Strokova, Irina Markova, Natalia Kozhukhova, Nikita Lukyanenko, Danil Potapov

**Affiliations:** 1 Department of Material Science and Material Technology, Belgorod State Technological University Named After V.G. Shukhov, 308012 Belgorod, Russia; sveta-zolotykh@yandex.ru (S.B.); vvstrokova@gmail.com (V.S.); irishka-31.90@mail.ru (I.M.); niklu03@gmail.com (N.L.); potapovdanil420@gmail.com (D.P.); 2 Department of Road Construction Materials and Chemical Technologies, Moscow Automobile and Road Construction State Technical University, 125319 Moscow, Russia; michaell1987@yandex.ru

## Addition of an Author

Mikhail Lebedev was not included as an author in the original publication [1]. The order of authors has been changed.

## Additional Affiliation

2. Department of Road Construction Materials and Chemical Technologies, Moscow Automobile and Road Construction State Technical University, 125319 Moscow, Russia

## Removal of Acknowledgement

The acknowledgements section has been removed.

## Author Contribution Correction

The corrected Author Contributions statement appears here:

**Author Contributions:** Conceptualization, M.L., V.S., and I.M.; methodology, S.B., M.L., and I.M.; software, N.K.; validation, N.L. and D.P.; formal analysis, S.B., M.L., and I.M.; investigation, S.B., M.L., I.M., N.L., and D.P.; resources, D.P.; data curation, S.B., N.K., N.L., and D.P.; writing—original draft preparation, S.B., M.L., and I.M.; writing—review and editing, V.S. and N.K.; visualization, S.B., M.L., and I.M.; supervision, V.S.; project administration, V.S. and I.M.; funding acquisition, V.S. All authors have read and agreed to the published version of the manuscript.

The authors state that the scientific conclusions are unaffected. This correction was approved by the Academic Editor. The original publication has also been updated.
